# Spectroscopic, DFT, and XRD Studies of Hydrogen Bonds in *N-*Unsubstituted 2-Aminobenzamides

**DOI:** 10.3390/molecules22010083

**Published:** 2017-01-04

**Authors:** Malose Jack Mphahlele, Marole Maria Maluleka, Lydia Rhyman, Ponnadurai Ramasami, Richard Mokome Mampa

**Affiliations:** 1 Department of Chemistry, College of Science, Engineering and Technology, University of South Africa, Private Bag X06, Florida 1710, South Africa; malulmm@unisa.ac.za (M.M.M.); p.ramasami@uom.ac.mu (P.R.); 2 Computational Chemistry Group, Department of Chemistry, Faculty of Science, University of Mauritius, Reduit 80837, Mauritius; lyd.rhyman@gmail.com; 3 Department of Chemistry, University of Limpopo, Private Bag X1106, Sovenga 0727, South Africa; Richard.Mampa@ul.ac.za

**Keywords:** halogenated 2-aminobenzamides, ^1^H-NMR spectra, vibrational spectra, DFT, XRD

## Abstract

The structures of the mono- and the dihalogenated *N*-unsubstituted 2-aminobenzamides were characterized by means of the spectroscopic (^1^H-NMR, UV-Vis, FT-IR, and FT-Raman) and X-ray crystallographic techniques complemented with a density functional theory (DFT) method. The hindered rotation of the C(O)–NH_2_ single bond resulted in non-equivalence of the amide protons and therefore two distinct resonances of different chemical shift values in the ^1^H-NMR spectra of these compounds were observed. 2-Amino-5-bromobenzamide (**ABB**) as a model confirmed the presence of strong intramolecular hydrogen bonds between oxygen and the amine hydrogen. However, intramolecular hydrogen bonding between the carbonyl oxygen and the amine protons was not observed in the solution phase due to a rapid exchange of these two protons with the solvent and fast rotation of the Ar–NH_2_ single bond. XRD also revealed the ability of the amide unit of these compounds to function as a hydrogen bond donor and acceptor simultaneously to form strong intermolecular hydrogen bonding between oxygen of one molecule and the NH moiety of the amine or amide group of the other molecule and between the amine nitrogen and the amide hydrogen of different molecules. DFT calculations using the B3LYP/6-311++G(d,p) basis set revealed that the conformer (**A**) with oxygen and 2-amine on the same side predominates possibly due to the formation of a six-membered intramolecular ring, which is assisted by hydrogen bonding as observed in the single crystal XRD structure.

## 1. Introduction

The amide moiety is abundant in biologically-relevant molecules and its propensity for hydrogen bonding plays an important role in the spatial structure of proteins, nucleic acids and biological membranes as well as in the interaction of bioactive compounds with receptors [1,2]. From a structural point of view, hydrogen bond formation causes changes in the distances between atoms and the rearrangement of electron densities on the groups involved in the interaction and therefore chemical reactivity (nucleophilicity or electrophilicity) especially if the reaction centres are directly involved in the hydrogen bonding [1,3]. Small model systems with intramolecular and/or intermolecular hydrogen bonded amide groups are often employed to study these interactions in solution and in the solid state [4]. Aminobenzamides, for example, are commonly used as hydrogen-bond donors to form intramolecular hydrogen bonds with other *O*- or *N*-groups as hydrogen-bond acceptors [5,6]. The amides also work as dual hydrogen-bond donors and acceptors to form intramolecular hydrogen bonds between the adjacent amide-amide groups to construct linear sheets and helical conformations [7,8,9]. The nature of the hydrogen-bonding network in the reagent, on the other hand, has been found to dictate not only what chemical intermediates will form, but also which polymorph of the product will be nucleated. 2-Acetamidobenzamide, for example, was found to undergo a series of thermal solid-state rearrangements in which a polymorphic transformation and cyclodehydration occur to give anhydrous 2-methylquinazolin-4-one [10].

2-Aminobenzamide (anthranilamide) and its derivatives are efficient direct Factor Xa (FXa) inhibitors, which plays a significant role in the blood coagulation cascade and catalyzes the conversion of prothrombin to thrombine [11,12]. These compounds are extensively used as fluorescence labels for the visualization of oligosaccharides after the cleavage from glycoprotein [13]. Oligomers derived from anthranilamide, on the other hand, exhibit intrinsic dipole moments which make them suitable candidates to serve as bioinspired molecular electrostatic analogues of magnets also known as electrets [14]. A series of novel bioactive mixed ligand Cu(II) complexes [15] and Ni(II) complexes [16] have been prepared before from 2-aminobenzamide and some amino acids and then evaluated for biological properties. 2-Aminobenzamide and its derivatives are envisaged to exist in four tautomeric forms and two closed pharmacophore sites through the formation of six-membered intramolecular rings assisted by hydrogen bonding, which are considered to be responsible for decreasing both antibacterial (C=O^δ−^–^δ+^HN) and antifungal activity (C-OH^δ+^–^δ−^N=C) [17]. 2-Aminobenzamide, on the other hand, is considered to lack intramolecular hydrogen bonds and this is attributed to a rapid exchange of the two protons of amine hydrogens with the solvent [18]. However, intramolecular hydrogen bonding has been observed for the corresponding *N*-aryl substituted anthranilamides in solution and in the solid state [18]. Based on this observation, the authors then concluded that a substituent on the nitrogen atom of the arylamine moiety plays an important role in promoting the formation of intramolecular hydrogen bonds for these compounds.

Despite the enormous pharmacological [11,12,13,15,16,17] and chemical [19,20,21,22,23,24] interest in the *N*-unsubstituted 2-aminobenzamides, to our knowledge, the presence of intramolecular hydrogen bonding in these compounds has been less demonstrated in the literature [25,26,27,28]. This prompted us to study the structural properties of the known 5-bromo/iodo- and 3,5-dihalogenated 2-aminobezamides by means of spectroscopic (NMR, UV-Vis, IR, Raman) methods and single X-ray crystallography in combination with density functional theory methods.

## 2. Results and Discussion

### 2.1. Synthesis

**Scheme 1:**
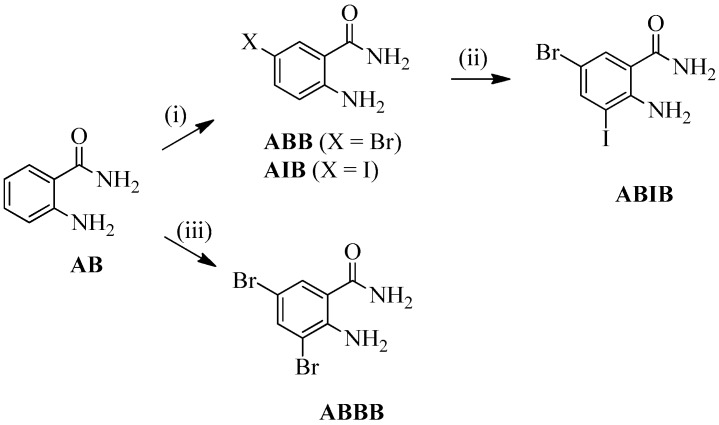
Halogenation of 2-aminobenzamide (**AB**). *Reagents & conditions*: (**i**) NBS, CH_3_CN, RT, 0.5 h (X = Br) or NIS, AcOH, RT, 1 h (X = I); (**ii**) NIS, AcOH, RT, 1 h; (**iii**) NBS (2 equiv.), AcOH, RT, 2 h.

2-Amino-5-bromobenzamide (**ABB**) [19], 2-amino-5-iodobenzamide (**AIB**) [20], 2-amino-3,5-dibromobenzamide (**ABBB**) [21,22,23], and 2-amino-5-bromo-3-iodobenzamide (**ABIB**) [24] were previously prepared from the commercially available 2-aminobenzamide (**AB**) as depicted in Scheme 1.

The structure of the four compounds under investigation (**ABB**, **AIB**, **ABBB**, and **ABIB**) was evaluated in solution by means of ^1^H-NMR and UV-Vis spectroscopy and in the solid state using vibrational (IR and Raman) spectroscopic methods. The structures were simulated in the gas and solution phases using the B3LYP/6-311++G(d,p) basis set or the LanL2DZ ECP basis set in the case of the iodine-containing derivatives. Single crystal X-ray diffraction (XRD), on the other hand, revealed the presence of intramolecular and intermolecular hydrogen bonding.

### 2.2. Solution Phase Studies Using ^1^H-NMR and UV-Vis Spectroscopy

**Figure 1:**
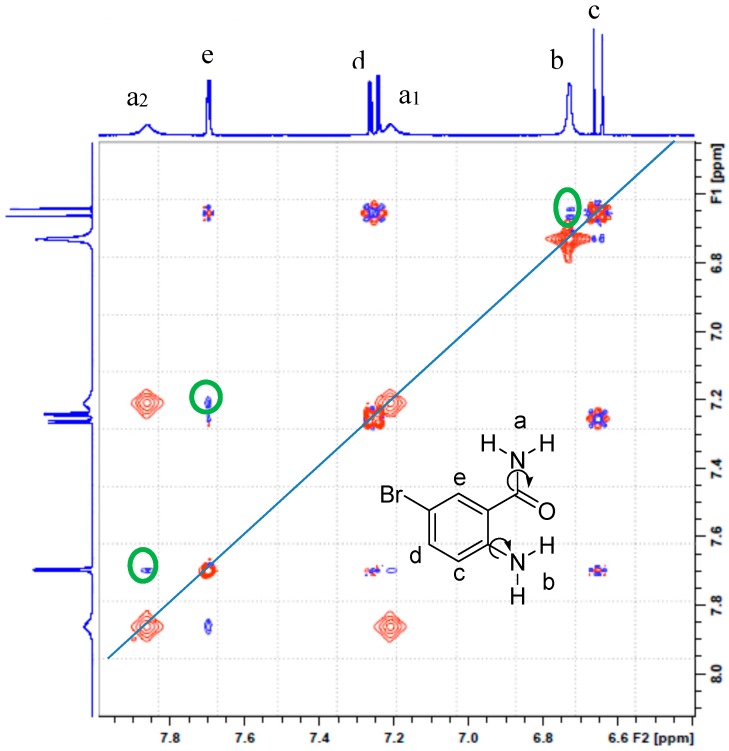
The 2D NOESY spectrum of **ABB** in DMSO-*d*_6_ at 400 MHz.

The ^1^H-NMR spectra of the 2-amino-5-bromobenzamide **ABB**, 2-amino-5-iodobenzamide **AIB**, 2-amino-3,5-dibromobenzamide **ABBB** and 2-amino-5-bromo-3-iodobenzamide **ABIB** acquired in DMSO-*d*_6_ at 500 MHz are consistent with the expected structures (refer to Supplementary material, S1, for full spectra). The spectra reveal the presence of the expected set of multiplets corresponding to the aromatic protons and three broad singlets in the aromatic region (δ_H_ 6.50–7.80 ppm). An intense singlet around δ 6.70 ppm in the spectra of **ABB**, **AIB**, and **ABIB**, which is broader and less intense in the case of **ABBB** integrate for two protons. The other two singlets of equal intensity each integrating for a single proton resonate around δ 7.15 and δ 7.81 ppm for **ABB** and **AIB** and these singlets resonate relatively down field at about δ 7.35 ppm and δ 8.00 ppm for the dihalogenated 2-aminobenzamides **ABBB** and **ABIB**, respectively. Gorobets et al. previously observed a similar set of non-equivalent proton signals in the ^1^H-NMR spectra of a series of *N*-aryl substituted benzamides in DMSO-*d*_6_ and assigned them to the amide protons [3]. These authors attributed the non-equivalence of these protons to be the consequence of the hindered rotation of the C(O)–NH_2_ single bond [3]. The amide protons of benzamide in DMSO-*d*_6_ solution, on the other hand, have also been found to be non-equivalent and to resonate as the two well-resolved singlets of different chemical shifts [19]. In order to distinguish between the hydrogens bonded to the amine and amide nitrogen atoms and to assign the three broad singlets accordingly, we performed a 2D NOESY experiment on compound **ABB** (Figure 1). The 2D NOESY experiment revealed that the set of non-equivalent broad singlets of reduced intensity interact strongly (green circles) with a doublet (**e**) at δ 7.69 ppm for 6-H, whereas the intense broad singlet (**b**) around δ 6.70 ppm interacts strongly with a doublet (**c**) at δ 6.65 ppm for 3-H. Based on the 2D NOESY experiment which provides 3D spatial hydrogen interaction, we assigned the set of non-equivalent signals to the amide nitrogen. Their non-equivalence is attributed to some hindered rotation of the C(O)–NH_2_ single bond in terms of NMR time scale in analogy with the literature precedents for the *N*-aryl substituted benzamides [3] and benzamide [19]. The amine protons, on the other hand, resonate as a singlet around δ 6.70 ppm due to a fast Ar–NH_2_ single bond rotation. The difference in line widths and intensities of this signal for the four compounds is presumably due to H/D exchange with the solvent molecule. We can thus conclude that a fast rotation around the Ar–NH_2_ single bond and a rapid exchange of the two hydrogens with the solvent account for the lack of intramolecular hydrogen bonding between oxygen and amine hydrogens in solution in analogy with the literature observation for the 2-aminobenzamide [18].

The 2D NOESY experiment in our view further suggests the preponderance in solution of the conformer (see structure insert in Figure 1) with H-6 and the amide nitrogen in alignment to result in the observed strong interaction between H-6 and amide protons. Next, we performed geometry optimization of the two possible conformers (**A** and **B**) of 2-amino-5-bromobenzamide **ABB** in the gas phase and also in solution (DMSO) by means of density functional theory methods as described below.

### 2.3. DFT Studies

**Figure 2:**
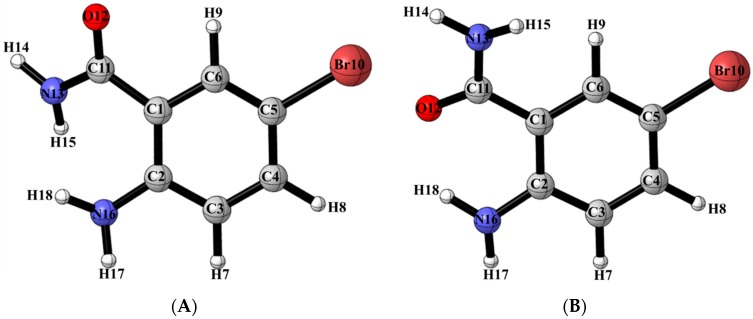
Geometry optimized conformers (**A**) and (**B**) of **ABB** in the solution phase.

The density functional theory (DFT) computations were carried out using the B3LYP exchange-correlation functional [29,30], together with the 6-311++G(d,p) [31] basis set for all atoms except for iodine in which case the LanL2DZ ECP basis set was used [32,33,34]. All computations were performed using the Gaussian 09 software suite [35] running on Gridchem [36,37]. The geometrical optimizations were performed in the gas phase as well as in dimethyl sulfoxide (DMSO). The solvent effect was taken into consideration based on the Polarizable Continuum Model (PCM) [38,39]. The tight criteria was used for the optimization and the frequency calculation was carried out to confirm the nature of the stationary points. In addition, the optimized structures in DMSO were used for computing chemical shifts with the Gauge-Including Atomic Orbital (GIAO) method [40] using shieldings of TMS computed at the same theoretical level and basis set. The TD-DFT [41,42,43] computation was conducted using the optimized structure in DMSO to obtain the electronic spectra. The DFT calculations using the B3LYP/6-311++G(d,p) basis set revealed that conformer (**A**) with oxygen and amine group in alignment is more stable than conformer (**B**) with amine and amide nitrogen aligned together by 15.8 kJ/mol and 9.4 kJ/mol in the gas phase and DMSO, respectively (Figure 2; Table 1). The free energy data and calculated equilibrium constant indicate that the percentage of conformer (**A**), which is implicated in Figure 1, is above 99%.

The computed GIAO ^1^H-NMR chemical shifts are collected in Table 2 (refer to Supplementary material, S2, for computed NMR data). The peculiar down-field shift of one of the hydrogen of the –NH_2_ group in conformer (**A**) is presumably the consequence of its involvement in the formation of six-membered intramolecular ring assisted by hydrogen bonding. Since protons attached to nitrogen are solvent and environment dependent, it is not easy to compare the theoretical values with the experimental ones. The computed chemical shift values are nevertheless in agreement with assignments of the experimental NMR spectra.

### 2.4. UV-VIS Spectroscopic Studies

**Figure 3:**
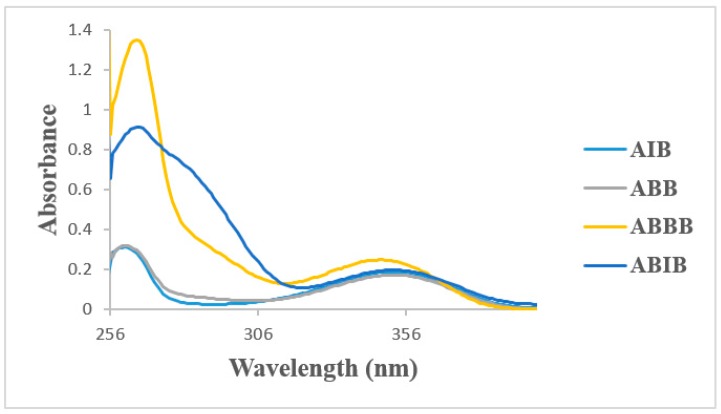
UV-Vis spectra of **ABB**, **AIB**, **ABBB**, and **ABIB** in DMSO (conc. = 1 × 10^−6^ mol/L).

**Figure 4:**
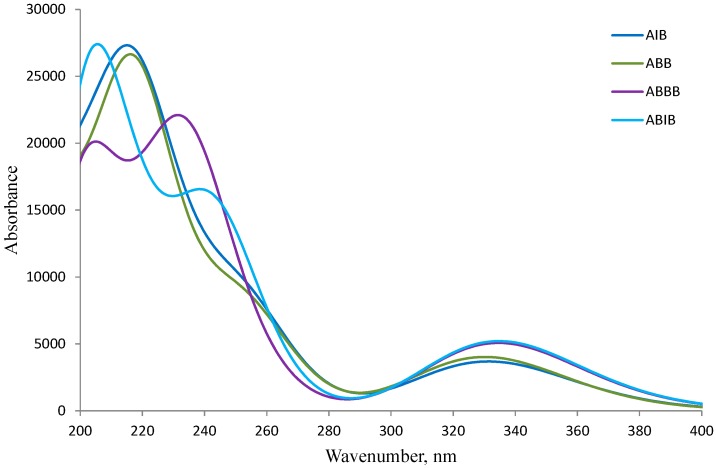
Simulated UV-Vis spectra of **ABB**, **AIB**, **ABBB**, and **ABIB** in DMSO.

The absorption spectra of the 2-aminobenzamides **ABB**, **AIB**, **ABBB** and **ABIB** (Figure 3) were acquired in DMSO at room temperature and are characterized by two discernible absorption bands, one around λ = 258 nm and the other around λ = 355 nm. The simulated absorption spectra of the 2-aminobenzamides in DMSO are illustrated in Figure 4 based on the TD-DFT computations. The difference between the calculated and the experimental spectra is due to the high polarity of DMSO which results in solvent-solute specific interactions. There is, however, a close similarity between the experimental and the simulated spectra for these compounds.

**Figure 5:**
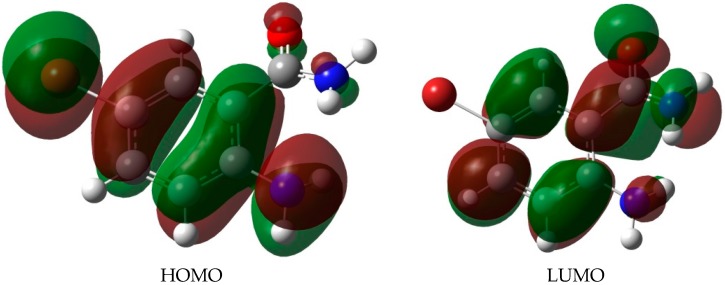
The HOMO and LUMO surfaces of **ABB** in DMSO.

A detailed analysis of the electronic transition of the 2-aminobenzamides in terms of frontier orbital energies, wavelength and oscillator strength was done by employing the TD-DFT/B3LYP calculations in both the gas phase and in DMSO solution (using IEF-PCM model) and the corresponding values are collected in Table 3 The electronic transition occurred between the frontier molecular orbitals from HOMO (highest occupied molecular orbitals) to LUMO (lowest unoccupied molecular orbitals) and the energy gap is about 4.60 eV. The HOMO and LUMO surfaces of the most stable conformer (**A**) of **ABB** are illustrated in Figure 5. The HOMO-LUMO energy gaps of these compounds demonstrate that the charge transfer interaction is taking place within the molecule towards the amide group. The strong charge transfer interaction through π-conjugated system results in substantial ground state Donor–Acceptor (DA) mixing and the appearance of a charge transfer band in the electron absorption spectrum.

Amides have the ability to undergo self-association in less or non-polar mediums such as chloroform and to form dimers in the solid state [44]. However, self-association has not been observed in the strongly polar solvents such as DMSO-*d*_6_ due to intermolecular hydrogen bonding to the solvent molecules [3]. This led us to investigate the title compounds in the solid state by means of IR and Raman spectroscopy as well as single crystal X-ray diffraction (XRD) technique.

### 2.5. Solid State Studies Using IR and Raman Spectroscopy

The experimentally determined IR frequencies of compounds **ABB**, **AIB**, **ABBB**, and **ABIB**, which are illustrated in Table 4 show characteristic peaks in the N-H stretching region ν 3157–3424 cm^−1^ due to amino (NH_2_) and amido (NH_2_CO) group vibrations. There is a good agreement between the experimentally determined vibrational spectra of these compounds (solid state) and those simulated using DFT method (refer to the Supplementary Materials for experimental IR spectra (Supplementary 3), and Supplementary 5 for computed IR frequencies). The computed IR data are uncorrected based on harmonic approximation. It is well known in the literature that on the basis of this approximation, the computed wavenumbers will be larger than the experimentally observed values because the experimental value is an anharmonic frequency whereas the calculated one is a harmonic frequency. The literature scale factor to have the corrected wavenumbers for the method used is 0.96. The proposed assignments are based on the experimental and the simulated IR spectral data of **ABB** as a model. The band at ν 3161 cm^−1^ corresponds to the stretching C-H vibration in benzene ring. The other three sets of bands corresponding to the N-H vibrations are assigned as follows: ν_as_(NH_2_) = 3395 cm^−1^, ν_s_(NH_2_) = 3355 cm^−1^, and ν(NH) = 3281 cm^−1^. The bands at ν = 1600 cm^−1^ and ν = 1672 cm^−1^ are attributable to the azomethine (ν_C=N_) and carbonyl (ν_C=O_) vibrations, respectively.

The calculated Raman frequencies for compounds **ABB**, **AIB**, **ABBB**, and **ABIB** (see Supplementary Materials, S5) also compare favourably with the experimental ones (refer to Supplementary 4, for the spectra). We have obtained single crystals of 2-amino-5-bromobenzamide (**ABB**) suitable for X-ray crystallography by slow evaporation of toluene. This compound then represented a model to study the solid state structure of these primary 2-aminobenzamides by X-ray crystallography as described below.

### 2.6. Solid State Studies of ABB Using X-ray Crystallography

**Figure 6:**
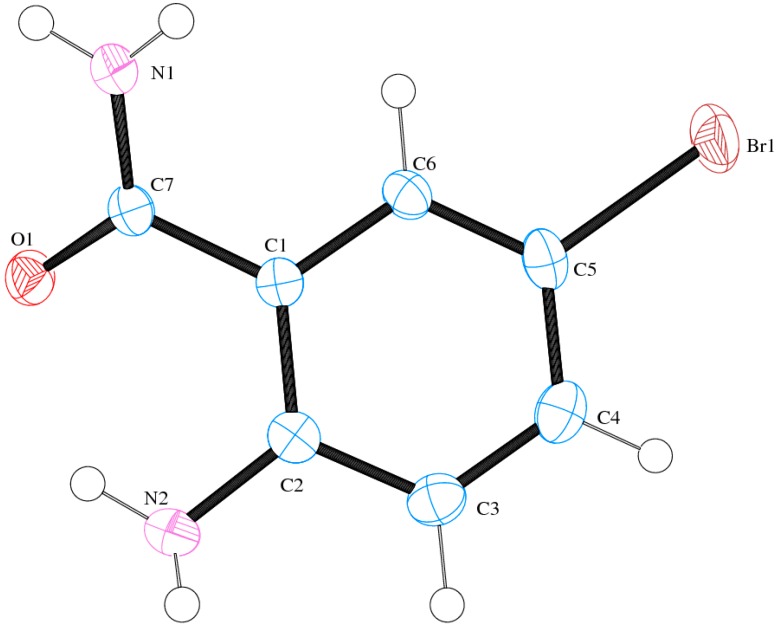
The Oak Ridge Thermal Ellipsoid Plot (ORTEP) diagram of **ABB** showing crystallographic labelling.

**Figure 7:**
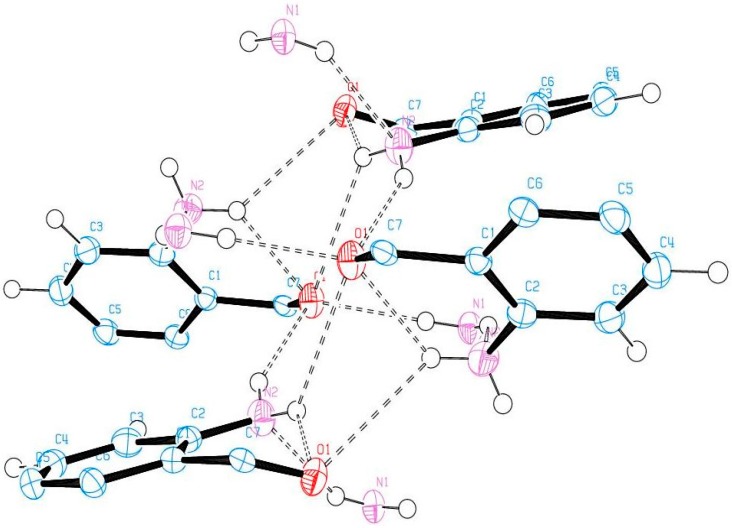
The X-ray crystal structure of **ABB** showing intra- and intermolecular hydrogen bonding.

Compound (**ABB**) crystallizes in the monoclinic space group C2/c (Figure 6; Table 5). Consideration of the atomic sizes suggests that deviation from co-planarity should occur in these molecules due to the presence of the heavy halogen atoms, which probably account for the high values for the largest diff peak [45]. We observed the intramolecular hydrogen bonds intuitively between oxygen of the amide moiety and NH of amine group (Figure 6). This X-ray crystal structure provides an unambiguous proof of the existence of intramolecular hydrogen bonding between the amide oxygen and the amine hydrogen for the *N*-unsubstituted 2-aminobenzamides in analogy with the literature precedents for 2-aminobenzamide [25] or 2-amino-3-chloro-5-nitrobenzamide [26] and the co-crystals involving 2-aminobenzamide [27,28]. XRD also revealed that the molecules are linked together by intermolecular hydrogen bonding. The amide unit of these compounds functioned as a hydrogen bond donor and an acceptor simultaneously to form an intermolecular hydrogen-bonded complex (Figure 7). There is strong intermolecular hydrogen bonding between oxygen of one molecule and the NH of the amine or amide group of the other molecule. Likewise, the amine nitrogen is involved in hydrogen bonding with an amide proton. These results are in agreement with the previous observation on the existence of intramolecular and intermolecular hydrogen bonding in 2-aminobenzamide [25] and 2-amino-3-chloro-5-nitrobenzamide [26].

## 3. Experimental

### 3.1. Spectroscopic Analysis

The ^1^H-NMR spectra for these compounds were obtained as DMSO-*d*_6_ solutions using Agilent 500 MHz NMR spectrometer (Agilent Technologies, Oxford, UK) and the chemical shifts are quoted relative to the TMS peak. The 2D NOESY experiment on compound **ABB** was performed on a Bruker 400 MHz NMR spectrometer (Bruker BioSpin GmhH, Karlsruhe, Germany) operating at 400 MHz (^1^H). The FT-IR spectra were recorded at room temperature as powders using a Bruker VERTEX 70 FT-IR Spectrometer (Bruker Optics, Billerica, MA, USA) with a diamond ATR (attenuated total reflectance) accessory by using the thin-film method. The Raman spectra were acquired on a Bruker Multiram FT-Raman spectrometer (Bruker Optics, Billerica, MA, USA) using 100 mW laser power with a resolution of about 4 cm^−1^ and 64 scans.

### 3.2. Single X-ray Data Collection and Processing

X-ray intensity data were determined on a Bruker Venture D8 Photon CMOS diffractometer (Bruker AXS, Madison, WI, USA) with graphite-monochromated MoKα_1_ (λ = 71073 Å) radiation at 173 K using an Oxford Cryostream 600 cooler. Data reduction was carried out using the program *SAINT+*, version 6.02 [46] and face-indexed [47] absorption corrections were made using the program *XPREP* [47]. Space group assignments was made using *XPREP* [47]. The structure was solved in the *WinGX* [47] suite of programs, using direct methods through using *SHELXS-97* [48] and refined using full-matrix least-squares/difference Fourier techniques on F^2^ using *SHELXL-97* [48]. Thereafter, the hydrogen atoms attached to the N atoms were located in the difference Fourier map and the coordinates and isotropic parameter refined freely. All C-H hydrogen atoms were placed at idealized positions and refined as riding atoms with isotropic parameters 1.2 times those of their parent atoms. Diagrams and publication material were generated using *ORTEP-3* [49] and *PLATON* [50].

## 4. Conclusions

The non-equivalence of the amide protons in the solution phase is due to a hindered rotation around the CO-NH_2_ single bond. Intramolecular hydrogen bonding between the carbonyl oxygen and the amine proton was not observed in the solution phase due to a rapid exchange of the two amine hydrogens with the solvent. The presence of strong intramolecular hydrogen bonds was, however, verified by the crystal structure of **ABB** in the solid state, which also revealed the presence of strong intermolecular hydrogen bonding between oxygen of one molecule and NH of the amine or amide group of the other molecule. Such interactions also exist between the amine nitrogen and the amide proton, and between the amine nitrogen and the amide hydrogen. Molecular orbital coefficient analyses suggest that the electronic spectrum corresponds to the π→π* electronic transition. There is a good agreement between experimentally determined structural parameters and the vibrational frequencies of the compounds and those predicted theoretically using the DFT method. Our results which incorporate X-ray data of the halogenated 2-aminobenzamides complement previously-reported spectroscopic and computational data of *N*-substituted aminobenzamides and further confirm the presence of intramolecular hydrogen bonding in the *N*-unsubstituted 2-aminobenzamides in the solid state.

## Figures and Tables

**Table 1:** Electronic energy of the conformers of the four studied compounds and their energy difference in the gas phase and in DMSO.

	**Gas Phase**			**DMSO**		
	**B3LYP**					
	**Electronic Energy (Hartree)**		**ΔE (kJ/mol)**	**Electronic Energy (Hartree)**		**ΔE (kJ/mol)**
	**Conformer (A)**	**Conformer (B)**		**Conformer (A)**	**Conformer (B)**	
**ABB**	−3030.994480	−3029.988460	15.81	−3030.006409	−3030.002821	9.42
**AIB**	−467.228178	−467.222193	15.72	−467.240222	−467.236665	9.34
**ABBB**	−5603.535596	−5603.529423	16.21	−5603.546023	−5603.541927	10.75
**ABIB**	−3040.768917	−3040.762660	16.43	−3040.779561	−3040.775398	10.93
	**Gibb Free Energy (Hartree)**		**ΔG (kJ/mol)**	**Gibb Free Energy (Hartree)**		**ΔG (kJ/mol)**
**ABB**	−3029.897495	−3029.891223	16.47	−3029.909949	−3029.906365	9.41
**AIB**	−467.132463	−467.126178	16.50	−467.144972	−467.141348	9.51
**ABBB**	−5603.451831	−5603.445371	16.96	−5603.462814	−5603.458735	10.71
**ABIB**	−3040.686568	−3040.679861	17.61	−3040.697530	−3040.693577	10.38
	**B3LYP-D3**					
	**Electronic Energy (Hartree)**		**ΔE (kJ/mol)**	**Electronic Energy (Hartree)**		**ΔE (kJ/mol)**
	**Conformer (A)**	**Conformer (B)**		**Conformer (A)**	**Conformer (B)**	
**ABB**	−3030.010097	−3030.004505	14.68	−3030.022089	−3030.018918	8.32
**AIB**	−467.244209	−467.238646	14.61	−467.256320	−467.253167	8.28
**ABBB**	−5603.554266	−5603.548575	14.94	−5603.564784	−5603.561176	9.47
**ABIB**	−3040.788022	−3040.782271	15.10	−3040.798761	−3040.795110	9.58
	**Gibb Free Energy (Hartree)**		**ΔG (kJ/mol)**	**Gibb Free Energy (Hartree)**		**ΔG (kJ/mol)**
**ABB**	−3029.912976	−3029.907178	15.22	−3029.925554	−3029.922248	8.68
**AIB**	−467.148366	−467.142508	15.38	−467.160869	−467.157580	8.64
**ABBB**	−5603.470457	−5603.464475	15.71	−5603.481694	−5603.477920	9.91
**ABIB**	−3040.705570	−3040.699515	15.90	−3040.716715	−3040.713097	9.50

**Table 2:** The computed GIAO ^1^H-NMR chemical shifts of conformers (**A**) and (**B**) for **ABB**, numbering as per Figure 2.

	ABB	
	Conformer (A)	Conformer (B)
**H7**	7.02	7.11
**H8**	7.46	7.51
**H9**	7.73	8.05
**H14**	5.17	5.49
**H15**	5.76	6.30
**H17**	4.30	4.06
**H18**	7.45	4.18

**Table 3:** Calculated electronic parameters of the two conformers of 2-aminobenzamides in DMSO.

λ (nm)	λ (eV)	Oscillator Strength	Assignment	% Contribution
**Conformer (A)**				
**ABB** (HOMO-LUMO gap = 4.38 eV)				
216.6	5.72	0.1174	HOMO-3→LUMO	31.6
330.2	3.75	0.0993	HOMO→LUMO	96.8
**AIB** (HOMO-LUMO gap = 4.36 eV)				
213.6	5.80	0.2280	HOMO-4→LUMO	21.0
333.0	3.72	0.0840	HOMO→LUMO	93.8
**ABBB** (HOMO-LUMO gap = 4.34 eV)				
234.9	5.28	0.3589	HOMO-1→LUMO	44.7
334.7	3.70	0.1252	HOMO→LUMO	96.7
**ABIB** (HOMO-LUMO gap = 4.35 eV)				
243.1	5.10	0.3372	HOMO→LUMO+2	65.7
334.9	3.70	0.1186	HOMO→LUMO	89.4
**Conformer (B)**				
**ABB** (HOMO-LUMO gap = 4.65 eV)				
217.9	5.69	0.5301	HOMO-2→LUMO	35.2
311.0	3.99	0.0800	HOMO→LUMO	95.2
**AIB** (HOMO-LUMO gap = 4.62 eV)				
218.5	5.68	0.4237	HOMO-4→LUMO	24.5
313.9	3.95	0.0758	HOMO→LUMO	92.5
**ABBB** (HOMO-LUMO gap = 4.58 eV)				
273.7	4.53	0.0758	HOMO-3→LUMO	8.4
390.9	3.17	1.5953	HOMO→LUMO	98.5
**ABIB** (HOMO-LUMO gap = 4.57 eV)				
239.1	5.19	0.4646	HOMO→LUMO+2	43.2
316.3	3.92	0.0936	HOMO→LUMO	69.6

**Table 4:**
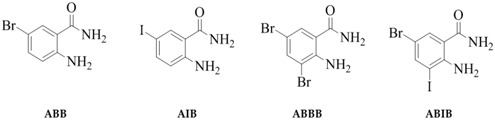
The experimental IR frequencies for compounds **ABB**, **AIB**, **ABBB**, and **ABIB**.

Compound	IR Frequencies
**ABB**	508, 534, 581, 652, 694, 818, 857, 1068, 1162, 1257, 1366, 1476, 1538, 1600, 1672, 3161, 3281, 3355, 3395
**AIB**	505, 537, 582, 657, 697, 798, 892, 1078, 1159, 1245, 1297, 1390, 1478, 1540, 1605, 1673, 3157, 3287, 3347, 3395
**ABBB**	413, 536, 584, 641, 800, 864, 1047, 1127, 1240, 1382, 1408, 1440, 1529, 1561, 1598, 1638, 3186, 3317, 3369, 3412
**ABIB**	413, 542, 644, 805, 873, 1054, 1130, 1240, 1276, 1386, 1414, 1451, 1536, 1567, 1602, 1641, 3180, 3326, 3368, 3424

**Table 5:** The crystal data and structure refinement for **ABB**.

CDCC	1511089	
Empirical formula	C_7_H_7_BrN_2_O	
Formula weight	215.06	
Crystal system	Monoclinic	
Space group	C2/c	
Unit cell dimensions	*a* = 30.3359(6) Å	α = 90°
	*b* = 6.50570(10) Å	β = 91.4270(10)°
	*c* = 7.7930(2) Å	γ = 90°
Volume	1537.52(6) Å^3^	
Z	8	
Density (calculated)	1.858 Mg/m^3^	
Absorption coefficient	5.285 mm^−1^	
F(000)	848	
Crystal size	0.44 × 0.19 × 0.06 mm^3^	
Theta range for data collection	1.34 to 27.99°	
Index ranges	−40 < h < 39, −8 < k < 8, −10 < l < 10	
Reflections collected	11742	
Independent reflections	1865 [R(int) = 0.0970]	
Completeness to theta = 27.99°	99.9%	
Absorption correction	Integration	
Max. and min. Transmission	0.7422 and 0.2045	
Refinement method	Full-matrix least-squares on F^2^	
Data/restraints/parameters	1865/0/116	
Goodness-of-fit on F^2^	1.018	
Final R indices [I > 2sigma(I)]	R^1^ = 0.0327, wR^2^ = 0.0854	
R indices (all data)	R^1^ = 0.0408, wR^2^ = 0.0879	
Largest diff. peak and hole	1.096 and −0.391 e.Å^−3^	

## Supplementary Materials

Supplementary materials can be accessed at: http://www.mdpi.com/1420-3049/22/1/83/s1. ^1^H-NMR, IR, and Raman spectra of compounds **ABB**, **AIB**, **ABBB**, and **ABIB** as well as their computed IR and Raman spectral data have been included as Supplementary Information.
