# Supplementary material for: Spectroscopic, DFT, and XRD Studies of Hydrogen Bonds in N-Unsubstituted 2-Aminobenzamides
Source: Molecules. 2017 Jan 4;22(1):83. doi: 10.3390/molecules22010083 (PMC6155760; doi:10.3390/molecules22010083)
Supplement: Supplementary file 1 [file molecules-22-00083-s001.pdf]

Supplementary Materials: Spectroscopic, DFT and XRD Studies of Hydrogen Bonds in *N*-Unsubstituted 2-Aminobenzamides

Malose Jack Mphahlele, Marole Maria Maluleka, Lydia Rhyman, Ponnadurai Ramasami and Richard Mokome Mampa

S1: ^{1}H-NMR spectra for ABB, AIB, ABBB and ABIB with peak pickings
S2: Computed GIAO NMR chemical shift of conformers (A) and (B) of ABB, AIB, ABBB and ABIB
S3: Experimental IR spectra for ABB, AIB, ABBB and ABIB
S4: Experimental Raman spectra for ABB, AIB, ABBB and ABIB
S5: Computed IR and Raman frequency values of ABB, AIB, ABBB and ABIB

1. Supplementary S1: Detailed ^{1}H-NMR Spectra for ABB, AIB, ABBB and ABIB

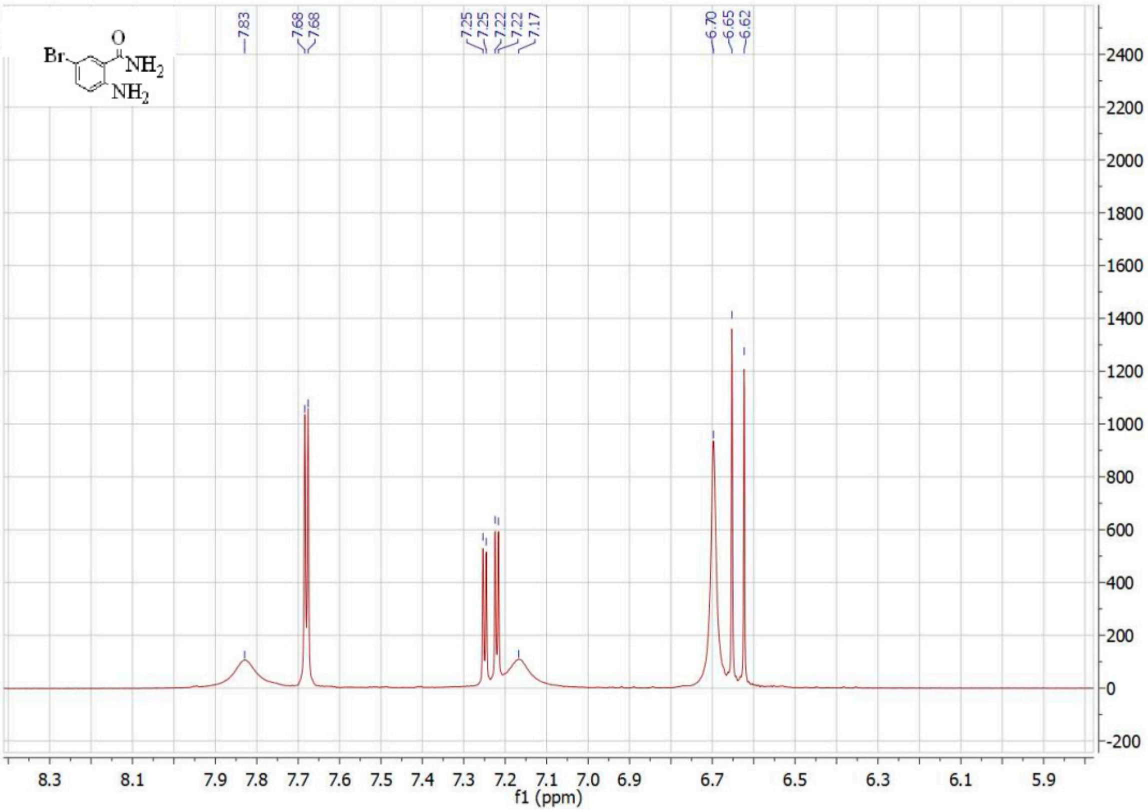

Figure S1. ^{1}H-NMR spectrum of **ABB** in DMSO-d_6 at 500 MHz showing peak pickings.

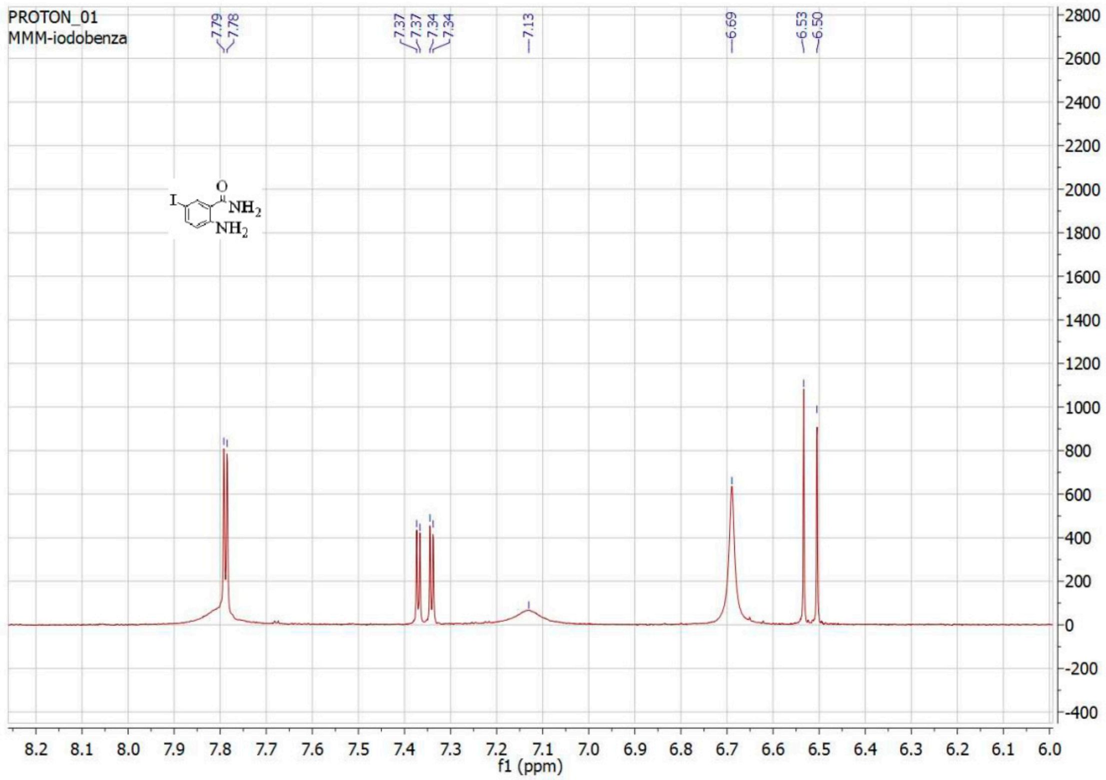

Figure S2. ^{1}H-NMR spectrum of **AIB** in DMSO-d_6 at 500 MHz showing peak pickings.

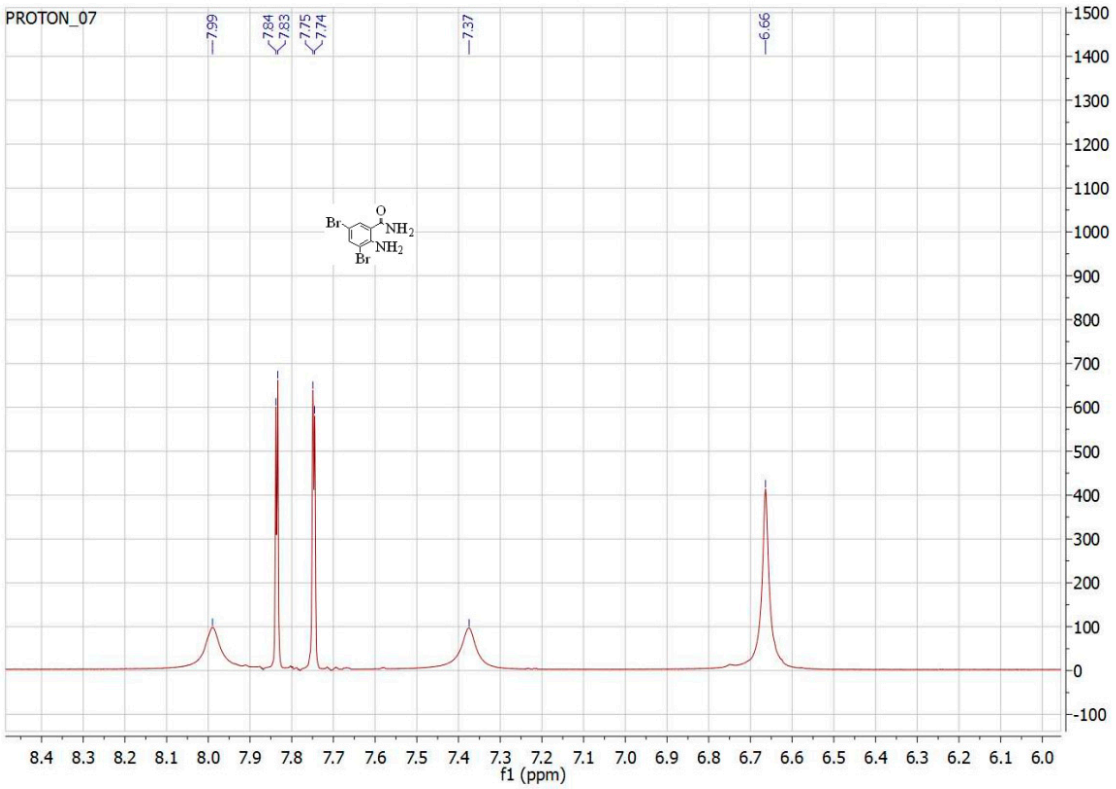

Figure S3. ^{1}H-NMR spectrum of **ABBB** in DMSO-d_6 at 500 MHz showing peak pickings.

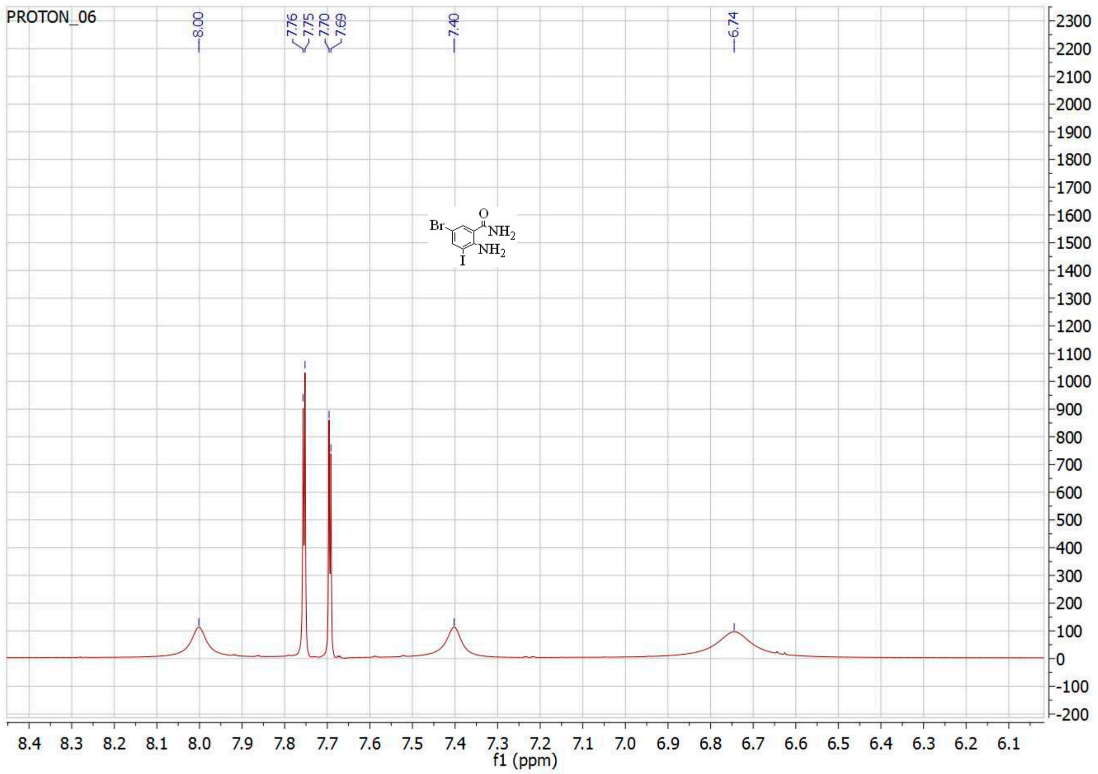

Figure S4. ^{1}H-NMR spectrum of **AIBB** in DMSO-d_6 at 500 MHz showing peak pickings.

2. Supplementary S2: Computed GIAO NMR Chemical Shift of Conformers (A) and (B) of ABB, AIB, ABBB and ABIB

Table S1. Computed chemical shift (ppm) of conformers (**A**) and (**B**) of **ABB**, **AIB**, **ABBB** and **ABIB**.

	**ABB**			
	**Conformer (A)**	**Conformer (B)**	**Conformer (A)**	**Conformer (B)**
C1	121.20	127.63	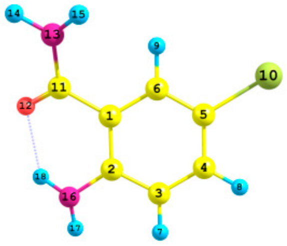	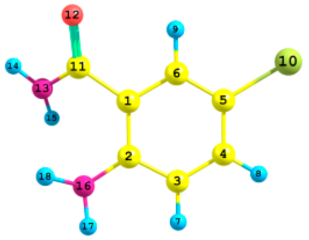
C2	157.76	153.77		
C3	124.18	124.83		
C4	142.90	142.31		
C5	129.99	134.00		
C6	137.47	140.26		
H7	7.02	7.11		
H8	7.46	7.51		
H9	7.73	8.05		
C11	178.27	175.06		
H14	5.17	5.49		
H15	5.76	6.30		
H17	4.30	4.06		
H18	7.45	4.18		
	**AIB**			
	**Conformer (A)**	**Conformer (B)**	**Conformer (A)**	**Conformer (B)**
C1	121.71	127.87		
C2	157.89	153.95		
C3	124.35	124.87		
C4	146.94	146.45		
C5	121.64	126.00		
C6	141.70	144.67		

H7	6.98	7.04	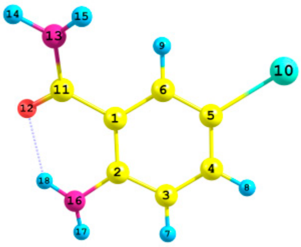	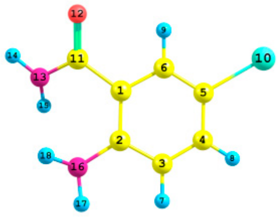
H8	7.46	7.53		
H9	7.78	8.09		
C11	178.23	175.18		
H14	5.12	5.46		
H15	5.70	6.29		
H17	4.20	3.98		
H18	7.35	4.15		

ABBB

	**Conformer (A)**	**Conformer (B)**	**Conformer (A)**	**Conformer (B)**
C1	145.08	144.58	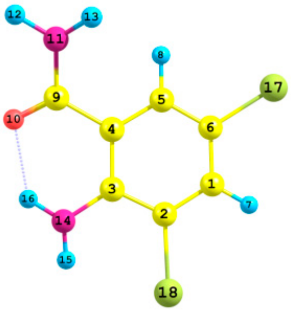	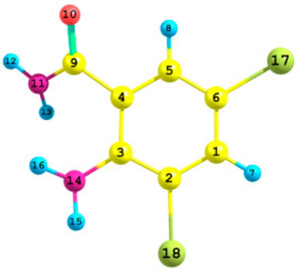
C2	132.99	133.38		
C3	154.55	150.52		
C4	122.75	128.84		
C5	137.32	140.51		
C6	129.53	132.73		
H7	7.73	7.75		
H8	7.63	7.90		
C9	177.31	174.11		
H12	5.26	5.56		
H13	5.75	6.00		
H15	4.87	4.80		
H16	7.97	4.70		

ABIB

	**Conformer (A)**	**Conformer (B)**	**Conformer (A)**	**Conformer (B)**
C1	122.80	129.11	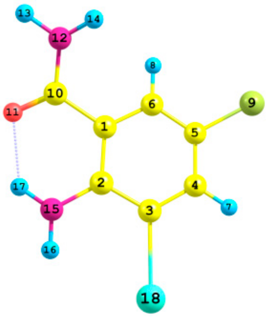	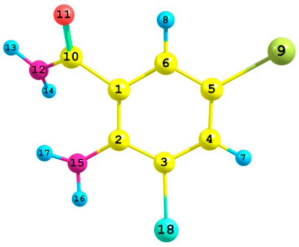
C2	156.59	152.79		
C3	127.87	128.18		
C4	149.52	148.89		
C5	129.70	132.76		
C6	138.04	140.74		
H7	7.86	7.89		
H8	7.75	7.89		
C10	177.61	174.77		
H13	5.27	5.60		
H14	5.74	6.01		
H16	4.67	4.53		
H17	7.95	4.70		

3. Supplementary S3: Experimental IR Spectra for ABB, AIB, ABBB and ABIB

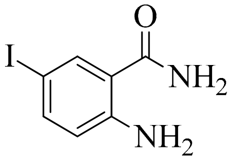

AIB

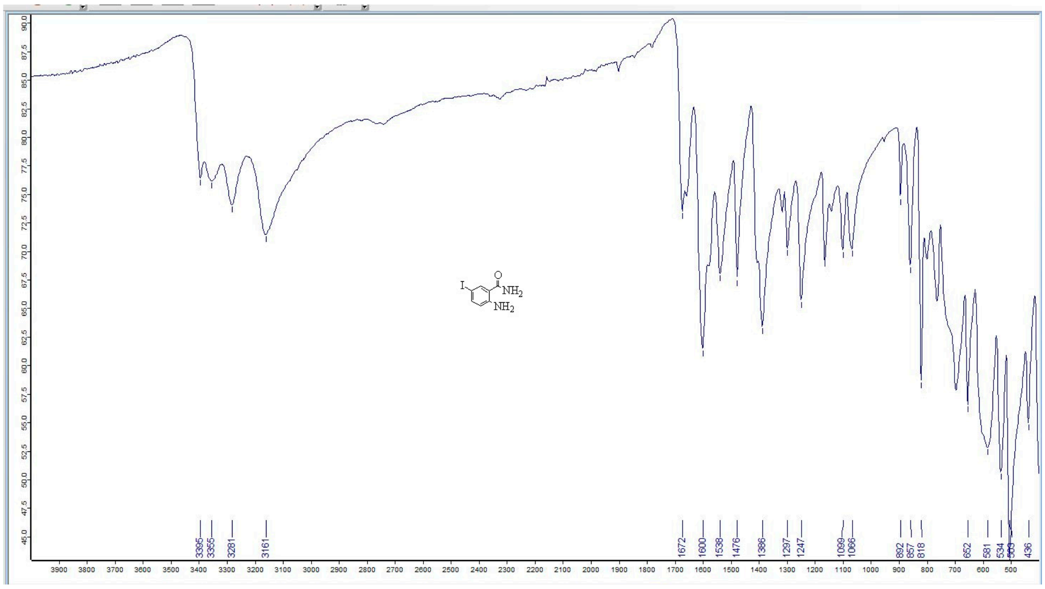

Figure S5. Experimental and computed IR spectra for **AIB**.

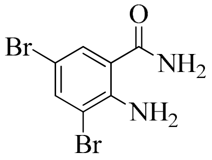

ABBB

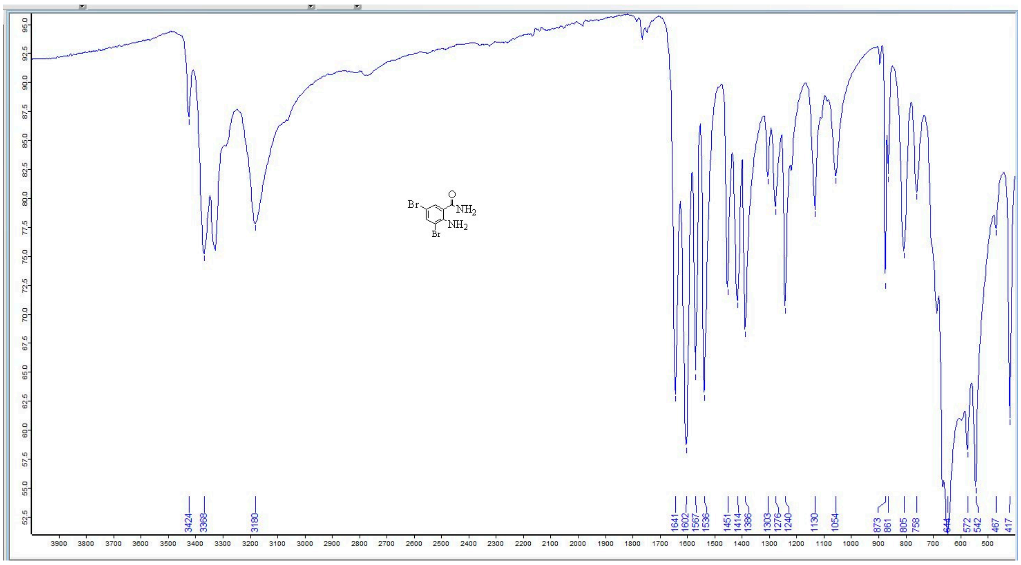

Figure S6. Experimental IR spectra for **ABBB**.

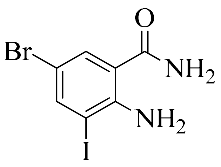

ABIB

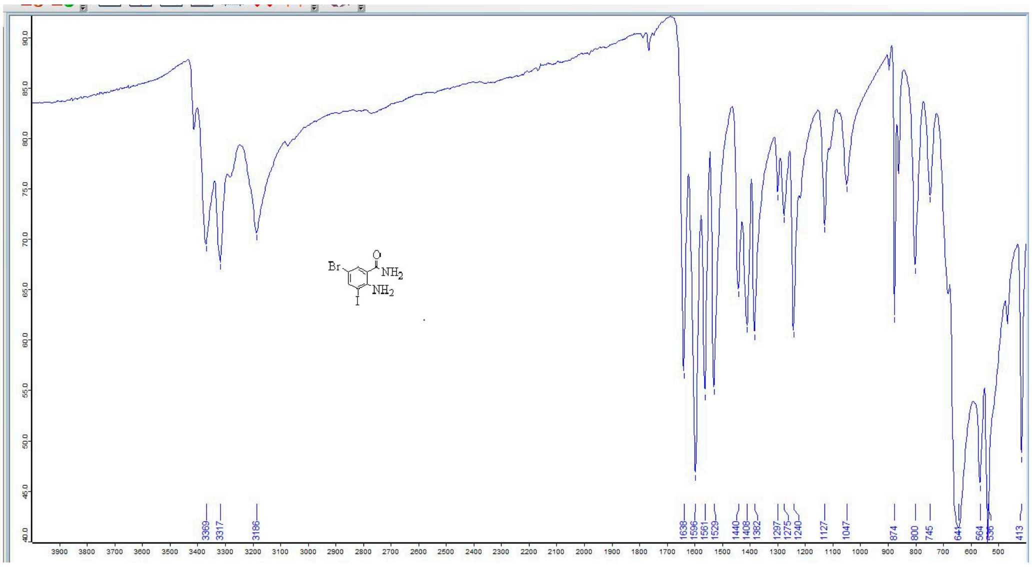

Figure S7. Experimental IR spectra for **ABIB**.

4. Supplementary S4: Experimental Raman Spectra for AIB, ABBB and ABIB

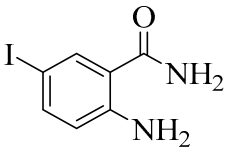

AIB

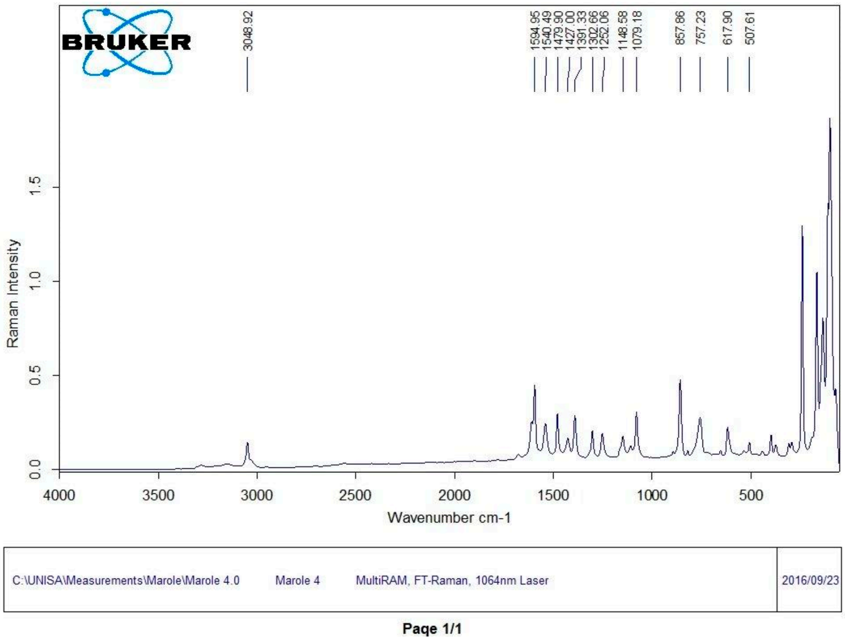

Figure S8. Experimental Raman spectrum of **AIB**.

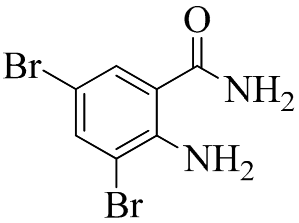

ABBB

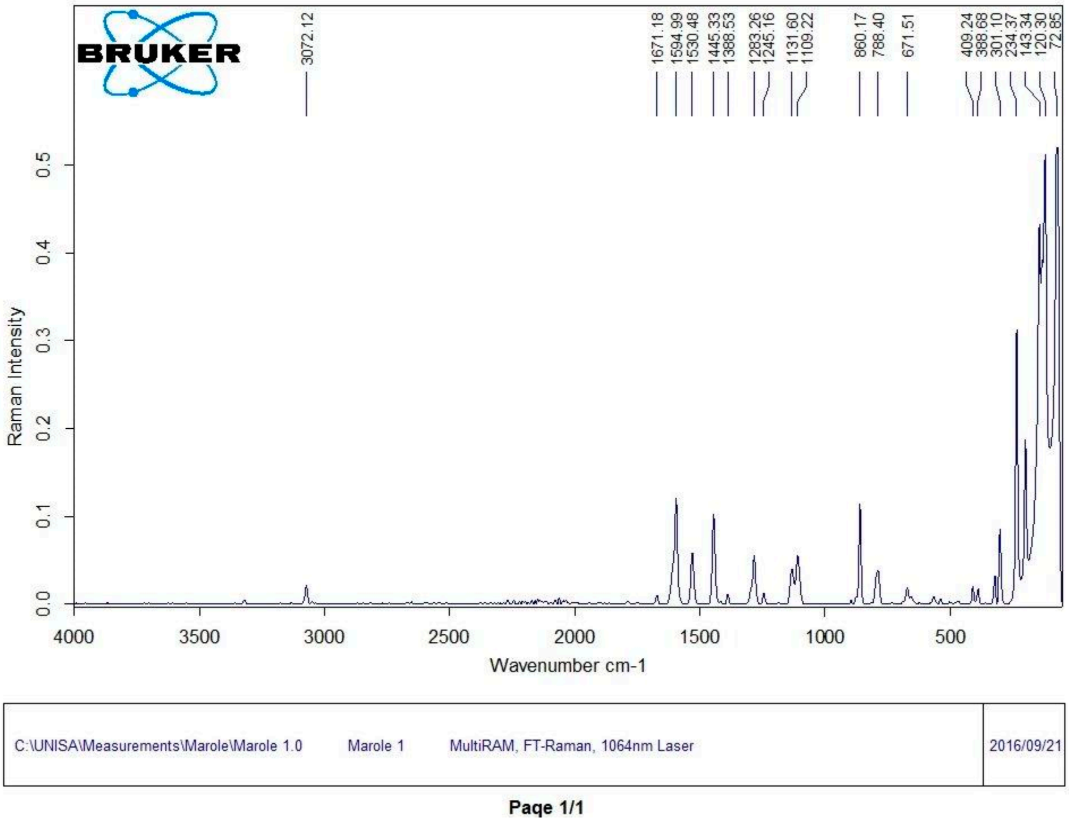

Figure S9. Experimental Raman spectrum of **ABBB**.

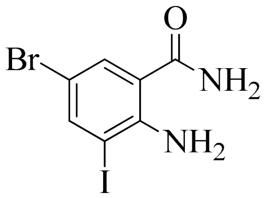

ABIB

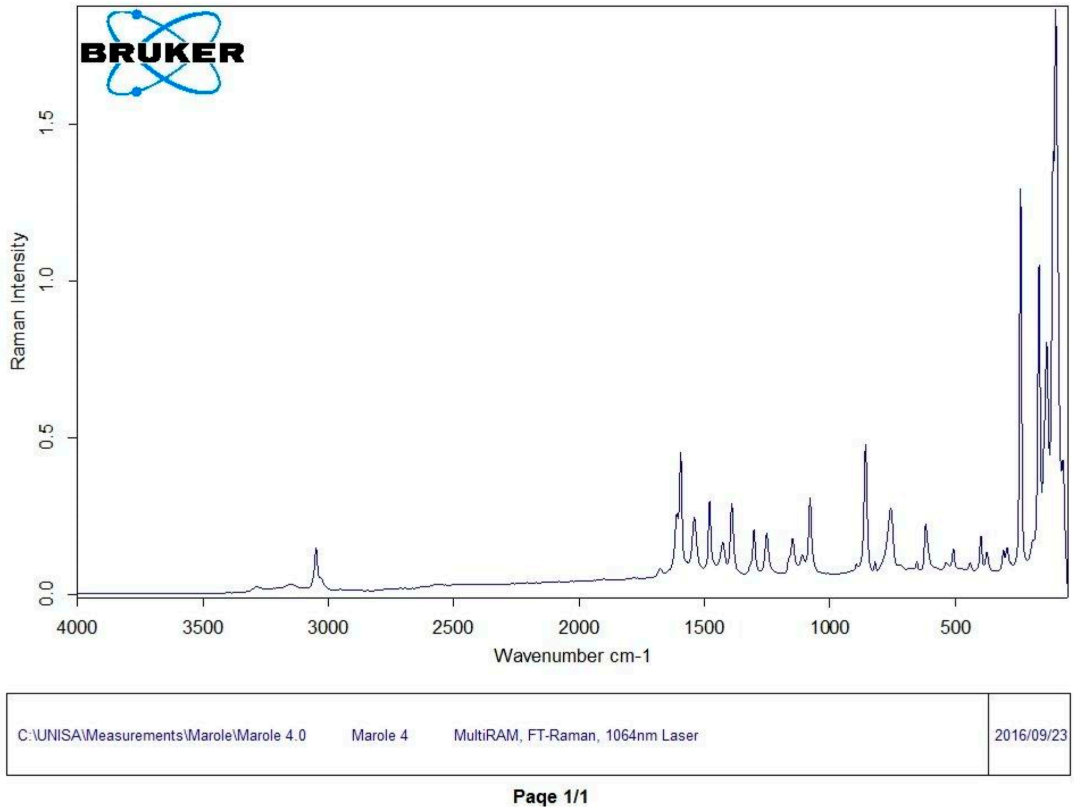

Figure S10. Experimental Raman spectrum of **ABIB**.

5. Supplementary S5: Computed IR and Raman Frequency Values and Intensities of ABB, AIB, ABBB and ABIB

Table S2. Computed IR frequency, IR intensity and Raman activity of conformer (**A**) for **ABB**.

Frequency (cm^{-1})	IR Intensity ($Km{\cdot}mol^{-1}$)	Raman Activity ($Å^4{\cdot}amu^{-1}$)	Selected Assignment
59.6	4.2	1.2	
110.3	0.6	1.5	
137.1	4.6	1.5	
144.6	1.0	1.7	
278.1	2.1	6.7	
293.0	11.7	1.9	
301.6	3.8	0.6	
360.6	201.5	1.4	
378.2	92.0	1.1	
385.0	69.4	2.5	
409.9	7.5	3.0	
431.6	5.9	1.9	
504.1	2.1	0.5	
530.2	4.4	0.3	

554.2	50.7	0.5	
617.7	81.8	1.2	
641.3	11.1	2.1	
651.7	39.4	4.7	
708.9	7.8	0.5	
755.9	6.1	18.3	
795.2	4.3	0.2	
827.5	38.8	0.7	
870.8	11.1	19.6	
900.9	6.7	0.3	
961.5	0.1	0.1	
1066.1	4.4	0.8	
1079.4	17.6	21.0	
1113.5	6.4	7.5	
1132.7	19.2	21.6	
1186.2	29.4	3.9	
1287.4	98.1	14.1	
1333.4	44.3	16.4	
1341.5	84.9	4.8	
1380.4	154.6	33.1	
1435.1	23.2	9.0	
1506.2	64.6	22.0	
1578.9	110.1	29.6	
1609.5	62.0	1.8	
1624.6	233.5	17.4	
1647.8	156.1	31.6	
1715.3	319.1	48.7	C=O str
3164.4	11.6	104.7	
3198.6	2.7	82.2	
3200.2	0.1	67.6	N-H antisym str
3524.7	129.7	166.6	N-H antisym str
3589.5	51.4	153.9	N-H sym str
3687.9	63.8	82.8	N-H sym str
3713.5	40.5	44.8	C=O str

Table S3. Computed IR frequency, IR intensity and Raman activity of conformer (**B**) for **ABB**.

Frequency (cm^{-1})	IR Intensity ($Km{\cdot}mol^{-1}$)	Raman Activity ($Å^4{\cdot}amu^{-1}$)	Selected Assignment
61.4	9.2	1.2	
113.5	0.4	1.0	
131.7	6.7	1.7	
143.0	3.6	1.3	
268.3	38.7	4.1	
274.9	2.6	1.1	
284.5	6.6	5.5	
312.6	7.8	2.5	
367.6	3.6	2.3	
375.6	2.3	2.3	
437.9	9.2	0.4	
514.2	36.3	0.7	
542.0	59.0	0.4	
562.7	39.2	5.0	
585.9	28.1	1.4	

617.5	132.8	1.9	
641.0	10.8	2.7	
705.4	19.9	2.2	
715.4	164.9	1.7	
763.2	3.5	15.7	
797.3	7.4	0.8	
826.4	47.5	0.5	
865.8	22.4	19.0	
930.7	6.9	0.2	
963.8	1.3	0.1	
1073.9	9.1	1.2	
1095.9	7.0	1.6	
1110.2	8.9	29.8	
1138.9	10.5	12.0	
1183.1	21.1	7.0	
1267.1	32.8	12.8	
1313.1	42.7	11.9	
1326.3	135.8	8.7	
1347.3	72.3	18.3	
1439.0	110.2	2.6	
1506.4	129.3	3.9	
1601.5	7.4	15.6	
1619.6	153.3	24.3	
1633.3	8.2	27.4	
1663.4	105.9	24.0	
1741.7	345.8	40.0	C=O str
3160.6	12.5	107.1	
3200.1	1.7	107.3	
3212.1	4.5	42.1	N-H antisym str
3545.1	43.9	290.8	N-H antisym str
3548.7	13.2	62.0	N-H sym str
3644.5	28.3	53.8	N-H sym str
3671.2	51.1	62.7	C=O str

Table S4. Computed IR frequency, IR intensity and Raman activity of conformer (**A**) for **AIB**.

Frequency (cm^{-1})	IR Intensity ($Km{\cdot}mol^{-1}$)	Raman Activity ($Å^4{\cdot}amu^{-1}$)	Selected Assignment
58.5	3.5	1.4	
102.9	1.2	1.9	
124.8	1.0	1.9	
135.1	4.2	1.5	
228.0	0.3	9.5	
284.3	14.9	1.1	
289.8	1.2	1.4	
361.3	185.2	1.5	
377.1	110.6	1.3	
385.5	64.2	2.2	
408.6	10.4	3.0	
429.7	6.3	1.7	
502.6	2.3	0.8	
528.6	5.9	0.8	
552.4	55.1	2.2	
611.8	66.8	2.5	

626.2	22.9	1.1	
649.9	38.4	3.5	
709.4	7.1	3.1	
751.0	4.5	17.9	
795.3	3.7	0.1	
825.4	36.4	1.4	
862.7	13.4	25.2	
899.1	3.8	0.8	
959.5	0.2	0.8	
1061.1	7.3	1.5	
1079.7	16.0	20.8	
1108.2	8.4	6.0	
1129.8	15.5	13.1	
1189.7	30.3	4.2	
1289.7	104.0	15.4	
1333.9	39.6	12.9	
1340.5	82.9	5.3	
1378.4	165.9	27.8	
1430.7	22.0	9.7	
1504.5	59.9	23.1	
1576.2	107.6	26.6	
1607.2	66.4	1.9	
1623.8	220.0	22.3	
1645.2	173.7	23.6	
1714.8	320.5	52.1	C=O str
3162.0	11.8	112.3	
3195.9	3.7	37.6	
3197.1	0.4	98.6	N-H antisym str
3525.3	130.7	167.4	N-H antisym str
3589.0	51.1	156.0	N-H sym str
3687.9	63.4	84.9	N-H sym str
3712.7	40.2	45.3	C=O str

Table S5. Computed IR frequency, IR intensity and Raman activity of conformer (**B**) for **AIB**.

Frequency (cm^{-1})	IR Intensity ($Km\cdot mol^{-1}$)	Raman Activity ($Å^4\cdot amu^{-1}$)	Selected Assignment
60.0	9.3	1.5	
106.6	0.8	1.1	
124.1	0.8	1.6	
131.3	8.9	1.7	
228.5	1.1	8.4	
268.1	40.0	2.6	
272.5	3.6	1.1	
303.5	10.1	3.4	
365.1	3.1	2.2	
374.6	2.1	2.1	
436.1	8.7	0.5	
512.5	40.0	1.1	
540.9	54.3	0.6	
562.2	47.8	5.1	
583.8	19.3	2.7	
604.1	126.2	1.8	
636.0	11.0	2.5	

707.2	44.8	3.1	
712.5	149.1	2.4	
758.8	3.9	14.3	
796.5	6.8	0.5	
823.1	44.5	0.7	
857.5	25.3	23.9	
930.6	4.4	1.3	
962.4	1.6	0.5	
1069.1	11.2	2.3	
1093.5	7.1	0.8	
1110.7	8.5	27.7	
1135.7	9.1	7.4	
1186.3	23.0	7.6	
1269.2	36.8	12.5	
1315.2	53.8	10.0	
1325.3	119.0	9.7	
1345.4	90.7	16.1	
1435.1	112.2	1.9	
1504.9	121.9	5.2	
1598.7	7.5	15.3	
1619.2	155.7	29.8	
1630.1	8.6	21.7	
1663.4	108.0	21.4	
1741.2	340.1	38.3	C=O str
3158.1	13.0	114.7	
3197.7	2.6	96.9	
3208.8	3.8	38.7	N-H antisym str
3545.3	44.4	299.4	N-H antisym str
3549.0	13.4	65.1	N-H sym str
3644.5	28.1	54.8	N-H sym str
3671.5	50.5	64.5	C=O str

Table S6. Computed IR frequency, IR intensity and Raman activity of conformer (**A**) for **ABBB**.

Frequency (cm^{-1})	IR Intensity (Km·mol^{-1})	Raman Activity (Å^{4}·amu^{-1})	Selected Assignment
55.5	3.3	1.8	
84.1	1.1	0.5	
113.9	0.4	1.3	
123.4	0.2	2.3	
151.9	1.3	1.0	
183.1	1.6	1.5	
252.0	2.7	9.2	
295.3	6.5	4.4	
319.9	2.2	0.3	
342.2	22.2	0.7	
371.5	189.8	0.6	
383.6	62.8	0.4	
400.6	61.4	0.7	
418.5	19.2	2.3	
461.8	9.4	0.7	
551.7	1.4	0.5	
555.9	30.8	0.6	
558.2	15.0	0.4	

636.7	38.1	0.3	
656.0	54.7	4.4	
688.5	60.1	4.0	
724.4	2.6	5.5	
795.8	9.0	9.8	
801.1	16.4	0.7	
869.1	11.8	32.5	
889.4	17.8	0.3	
911.9	0.6	0.4	
1077.9	75.9	1.9	
1083.0	4.5	23.4	
1127.2	8.2	3.4	
1137.7	5.7	18.1	
1271.5	77.7	1.6	
1308.2	104.7	27.4	
1334.3	45.4	5.8	
1374.2	189.3	23.0	
1430.6	6.5	0.9	
1467.2	92.6	50.9	
1558.1	107.8	22.6	
1597.2	95.9	3.0	
1620.7	193.2	18.3	
1639.9	176.5	27.5	
1715.6	288.9	44.1	C=O str
3205.8	0.1	37.6	
3213.7	1.1	50.7	
3518.3	145.6	144.7	N-H antisym str
3589.1	58.5	168.9	N-H antisym str
3679.1	106.5	30.3	N-H sym str
3713.2	42.4	48.1	N-H sym str

Table S7. Computed IR frequency, IR intensity and Raman activity of conformer (**B**) for **ABBB**.

Frequency (cm^{-1})	IR Intensity ($Km{\cdot}mol^{-1}$)	Raman Activity ($Å^4{\cdot}amu^{-1}$)	Selected Assignment
56.4	11.0	1.4	
88.7	0.6	0.5	
116.7	0.8	1.1	
123.1	0.6	2.1	
149.7	1.5	0.9	
178.5	9.6	1.6	
251.8	1.2	10.2	
292.9	10.6	2.7	
303.3	44.7	4.6	
320.8	9.4	1.7	
331.8	3.5	0.4	
387.8	0.9	0.8	
388.7	6.6	1.3	
468.4	9.7	0.4	
537.3	68.9	1.3	
559.2	30.9	0.5	
580.5	40.5	0.9	
597.8	23.8	1.0	
620.8	105.2	1.1	

677.9	146.8	4.7	
702.9	56.0	4.5	
726.7	4.9	0.6	
790.8	14.8	2.2	
800.1	11.9	4.1	
864.4	16.8	29.5	
890.5	17.0	0.1	
936.0	3.7	0.1	
1085.6	56.3	2.4	
1102.8	8.1	5.7	
1123.5	0.5	17.9	
1138.8	9.2	12.4	
1260.0	29.3	2.3	
1289.8	17.7	25.5	
1319.2	119.9	1.5	
1342.2	111.5	12.7	
1429.1	23.0	8.7	
1470.6	249.7	4.2	
1577.2	10.5	15.3	
1612.1	75.5	36.3	
1624.0	88.2	3.3	
1657.5	122.4	28.5	
1745.1	345.9	39.2	C=O str
3215.1	1.5	48.8	
3215.8	5.4	43.2	
3545.7	45.4	240.7	N-H antisym str
3550.6	21.8	84.2	N-H antisym str
3646.2	61.4	24.9	N-H sym str
3672.9	50.6	63.9	N-H sym str

Table S8. Computed IR frequency, IR intensity and Raman activity of conformer (**A**) for **ABIB**.

Frequency (cm^{-1})	IR Intensity ($Km\cdot mol^{-1}$)	Raman Activity ($Å^4\cdot amu^{-1}$)	Selected Assignment
50.6	3.2	2.1	
77.6	1.0	0.6	
108.2	0.0	2.5	
113.1	0.4	1.3	
139.3	1.4	1.1	
174.4	2.1	1.9	
215.1	1.9	8.8	
290.9	2.9	5.5	
317.0	3.7	0.7	
332.1	13.6	0.7	
350.4	223.8	0.8	
374.8	25.5	0.4	
398.1	70.2	1.1	
417.3	10.3	2.5	
458.2	9.0	1.2	
547.7	0.2	0.8	
555.0	2.4	0.3	
556.0	49.6	1.4	
635.4	43.0	0.2	
656.4	54.1	3.7	

670.7	65.3	5.2	
729.7	0.9	9.4	
782.9	1.8	7.7	
803.0	24.4	2.8	
868.2	10.7	36.0	
890.9	14.3	0.9	
912.5	1.1	0.6	
1067.3	71.2	6.9	
1083.7	12.4	24.9	
1125.1	9.5	3.8	
1137.3	3.1	15.1	
1274.1	84.1	2.5	
1308.5	103.9	23.6	
1331.1	29.6	12.6	
1372.3	206.7	19.9	
1428.2	8.8	2.1	
1462.6	91.8	51.6	
1553.1	94.8	25.5	
1597.7	85.6	4.3	
1620.9	198.7	18.6	
1639.0	203.7	28.5	
1715.3	269.1	41.6	C=O str
3206.0	0.2	38.6	
3211.2	1.2	43.6	
3512.2	146.0	149.5	N-H antisym str
3588.4	59.2	174.4	N-H antisym str
3673.9	109.0	29.8	N-H sym str
3712.3	42.1	50.1	N-H sym str

Table S9. Computed IR frequency, IR intensity and Raman activity of conformer (**B**) for **ABIB**.

Frequency (cm^{-1})	IR Intensity ($Km \cdot mol^{-1}$)	Raman Activity ($Å^4 \cdot amu^{-1}$)	Selected Assignment
54.3	10.5	1.4	
83.3	1.0	0.7	
108.7	0.0	2.5	
115.3	1.0	1.2	
139.3	1.5	0.9	
169.0	11.7	2.1	
214.7	0.0	9.9	
287.4	22.3	4.1	
294.7	33.5	4.7	
319.8	9.8	1.7	
325.7	2.4	0.3	
382.4	1.0	0.8	
389.3	8.1	2.0	
466.6	7.6	0.2	
527.4	69.2	1.4	
556.1	24.3	0.7	
580.7	54.7	1.6	
591.5	19.4	1.3	
621.0	113.5	1.2	
665.5	114.0	7.4	
698.4	80.4	3.5	

732.1	0.8	2.3	
784.6	3.9	3.3	
801.5	22.2	1.7	
863.5	15.5	31.7	
891.3	12.0	0.4	
938.7	4.5	0.2	
1074.7	59.0	6.7	
1103.3	12.2	5.1	
1122.4	0.3	18.3	
1137.1	7.0	11.4	
1262.2	27.9	4.5	
1289.4	13.3	25.7	
1316.2	102.9	2.0	
1340.1	133.6	11.0	
1426.4	18.9	11.0	
1466.1	249.5	3.2	
1571.8	14.5	17.9	
1610.0	77.1	40.6	
1623.4	93.5	3.9	
1660.4	119.3	28.7	
1744.4	346.6	40.6	C=O str
3213.4	1.5	42.1	
3216.4	5.3	43.6	
3543.0	36.3	226.6	N-H antisym str
3550.7	26.2	112.5	N-H antisym str
3642.3	64.2	22.0	N-H sym str
3673.5	51.6	66.7	N-H sym str
